# All disease begins in the (leaky) gut: role of zonulin-mediated gut permeability in the pathogenesis of some chronic inflammatory diseases

**DOI:** 10.12688/f1000research.20510.1

**Published:** 2020-01-31

**Authors:** Alessio Fasano

**Affiliations:** 1 Mucosal Immunology and Biology Research Center, Center for Celiac Research and Treatment and Division of Pediatric Gastroenterology and Nutrition, Massachusetts General Hospital for Children, Boston, Massachusetts, USA; 2 European Biomedical Research Institute of Salerno, Salerno, Italy

**Keywords:** Chronic inflammatory diseases, Gut permeability, microbiome, zonulin

## Abstract

Improved hygiene leading to reduced exposure to microorganisms has been implicated as one possible cause for the recent “epidemic” of chronic inflammatory diseases (CIDs) in industrialized countries. That is the essence of the hygiene hypothesis that argues that rising incidence of CIDs may be, at least in part, the result of lifestyle and environmental changes that have made us too “clean” for our own good, so causing changes in our microbiota. Apart from genetic makeup and exposure to environmental triggers, inappropriate increase in intestinal permeability (which may be influenced by the composition of the gut microbiota), a “hyper-belligerent” immune system responsible for the tolerance–immune response balance, and the composition of gut microbiome and its epigenetic influence on the host genomic expression have been identified as three additional elements in causing CIDs. During the past decade, a growing number of publications have focused on human genetics, the gut microbiome, and proteomics, suggesting that loss of mucosal barrier function, particularly in the gastrointestinal tract, may substantially affect antigen trafficking, ultimately influencing the close bidirectional interaction between gut microbiome and our immune system. This cross-talk is highly influential in shaping the host gut immune system function and ultimately shifting genetic predisposition to clinical outcome. This observation led to a re-visitation of the possible causes of CIDs epidemics, suggesting a key pathogenic role of gut permeability. Pre-clinical and clinical studies have shown that the zonulin family, a group of proteins modulating gut permeability, is implicated in a variety of CIDs, including autoimmune, infective, metabolic, and tumoral diseases. These data offer novel therapeutic targets for a variety of CIDs in which the zonulin pathway is implicated in their pathogenesis.

## Introduction

Twenty-five hundred years ago, when Hippocrates stated that “All disease begins in the gut”, he had an incredible intuition that only recently has been fully appreciated because of new insights into the pathogenesis of many chronic inflammatory diseases (CIDs) afflicting humankind. Until 30 years ago, when the Human Genome Project was still in its planning stage, the general hypothesis was that genetic predisposition and exposure to an environmental trigger were both necessary and sufficient to develop CIDs, including infectious, allergic, neuroinflammatory/neurodegenerative, autoimmune diseases, and cancer. However, the epidemiological observation showing a major surge of CIDs during the past four decades in the Western hemisphere coincident with the declining rate of infectious diseases was at odds with the gene/environment paradigm ^1, 2^. This generated the hygiene hypothesis supporting the notion that we had made ourselves too clean for our own good and that people embracing a Western lifestyle would slowly die of CIDs instead of rapidly succumbing to infectious diseases as still is happening in developing countries.

**Figure 1.**
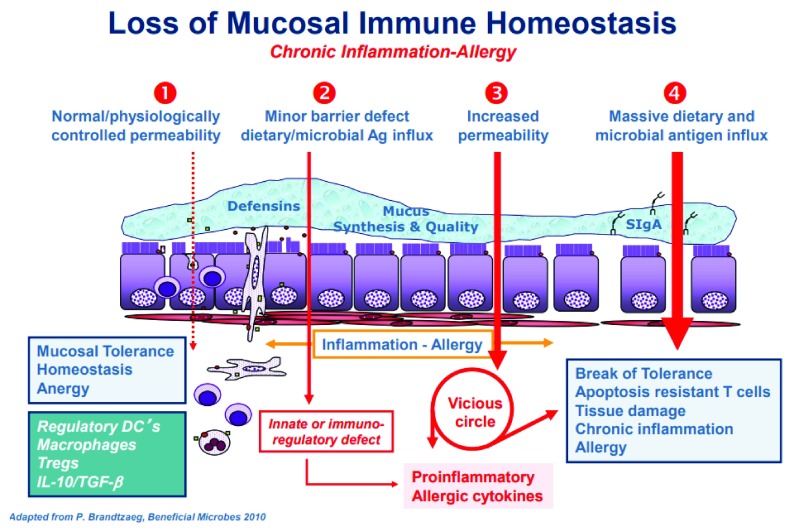
Proposed chain of events leading to chronic inflammatory disease. ( **1**) Under physiological circumstances there is a tightly control of mucosal antigen trafficking (antigen sampling) that, in concert with specific immune cells and chemokine and cytokine mediators lead to anergy and therefore to mucosal tolerance. ( **2**) Gut dysbiosis causes inappropriate production of increased amount of zonulin with subsequent functional loss of gut barrier function, followed by microbiota-derived antigen and endotoxin trafficking from the lumen to the lamina propria triggering innate and immunoregulatory responses causing a pro-inflammatory micromilieu. ( **3**) If this process continues, an adaptive immune response is mounted, causing production of pro-inflammatory cytokines, including interferon gamma (IFN-γ) and tumor necrosis factor alpha (TNF-α) that cause further opening of the paracellular pathway to the passage of antigens, creating a vicious cycle. ( **4**) Ultimately, these processes lead to break of tolerance with subsequent onset of chronic inflammatory disease whose nature is influenced by the specific host genetic background that dictates which organ or tissue will be targeted by the inflammatory process. DC, dendritic cell; IL-10, interleukin 10; TGF-β, transforming growth factor beta; Tregs, regulatory T cells. This figure was re-used from **Zonulin, a regulator of epithelial and endothelial barrier functions, and its involvement in chronic inflammatory diseases**. Tissue Barriers. doi:10.1080/21688370.2016.1251384 with permissions ^17^.

What we learned when the Human Genome Project was completed is that we are genetically much more rudimentary than we had previously thought. The premise of “one gene, one protein, one disease” cannot explain the complexity of the balance between health and disease and, most definitively, the CIDs epidemics. Twenty-three thousand genes are insufficient to explain all the permutations of human pathophysiology, including if and when and why we develop diseases. Rather, it is the interplay between us as individuals and the environment in which we live that dictates our clinical destiny. This interplay is physically and mechanistically regulated by biological interfaces that divide our body from the surrounding environment. At about 8 to 9 meters in length, the human intestine provides the largest interface between our body and the outside world. Tightly packed single layers of epithelial cells cover the external surfaces of our intestinal mucosa and negotiate the interaction with the surrounding environment. Although this enormous mucosal interface (200 m ^2^) is not apparently visible, it plays a pivotal role through its dynamic interactions with a variety of factors coming from our surrounding environment, including microorganisms, nutrients, pollutants, and other materials. Intestinal permeability, together with luminal antigen (Ag) sampling by enterocytes via the transcellular pathway and dendritic cells, regulates molecular trafficking between the intestinal lumen and the submucosa, leading to either tolerance or immune response to non-self Ag ^3– 5^ ( Figure 1). Intercellular tight junctions (TJs) tightly regulate paracellular Ag trafficking. TJs are extremely dynamic structures that operate in several key functions of the intestinal epithelium under both physiological and pathological circumstances ^6– 8^. TJs, the most apical junctional complex of the paracellular pathway that segregates the apical and basolateral cellular compartment, were previously believed to be impermeable and static, so forming a sealing barrier. This paradigm was subverted in 1993 by the discovery of zonula occludens 1 (ZO-1) as the first component of the TJ complex ^9^ now being comprised of more than 150 proteins, including occludin ^10^, claudins ^11^, junctional adhesion molecules (JAMs) ^12^, tricellulin ^13^, and angulins ^14^. However, despite major progress in our knowledge on the composition and function of the intercellular TJ, the mechanisms by which they are regulated are still incompletely understood. One of the major breakthroughs in understanding the role of gut permeability in health and disease has been the discovery of zonulin, the only physiologic intestinal permeability modulator described so far ^15, 16^. Therefore, this article will focus mainly on the body of literature published on zonulin as a biomarker of gut permeability to outline the pathogenic role of a leaky gut in a variety of CIDs. However, it should be pointed out that zonulin is not involved in all CIDs and that not all CIDs have been linked to increased gut permeability.

## The zonulin pathway and its activation

Zonulin is composed of a family of related proteins ^17– 19^ whose first member, pre-haptoglobin 2 (HP2), the precursor of HP2, was identified almost two decades ago ^20, 21^. Haptoglobins evolved from a complement-associated protein (mannose-binding lectin-associated serine protease, or MASP) that lost its protease function because of mutations in the catalytic domain to then acquire new functions, including the capability to modulate intercellular TJs ^18^. The frequent zonulin polymorphisms secondary to high mutation rate during evolution led to a family of structurally and functionally related zonulins, including pre-HP2 and properdin, another member of the MASP family ^22^.

Among the several potential intestinal luminal stimuli that can stimulate zonulin release, small exposure to large amounts of bacteria (bacteria overgrowth) and gluten, the protein causing celiac disease (CD), have been identified as the two most powerful triggers ^23, 24^. Zonulin secretion has been shown to be MyD88-dependent ^25^ and is followed by an increase in gut permeability secondary to the disassembly of the protein ZO-1 from the tight junctional complex ^26^. Gliadin triggers zonulin release through the CXCR3 receptor activated by its engagement to MyD88 with a subsequent increase in gut permeability ^27^, suggesting that gluten is misinterpreted by the zonulin pathway as a potential harmful component of a microorganism. Taken together, these data suggest that the activation of the zonulin pathway may represent a defensive mechanism that “flushes out” microorganisms, contributing to the innate immune response of the host against changes in microbiome ecosystem, specifically bacterial colonization of the small intestine (small intestinal bacterial overgrowth) or changes in its composition (dysbiosis) or both. These findings are in line with the growing evidence on the role of changes in gut microbiome composition and function in causing functional changes in gut permeability, with subsequent increased Ag trafficking and break of tolerance leading to CID in genetically susceptible individuals ( Figure 1).

## Pathological consequences of the activation of the zonulin pathway

Modulation of intestinal permeability, including the activation of the zonulin pathway, is part of the physiological machinery to maintain mucosal homeostasis and therefore does not always translate in clinical pathological outcomes. Indeed, studies on transgenic mice with constitutive activation of myosin light-chain kinase (MLCK), an intracellular mediator of TJ disassembly, showed increased intestinal permeability without signs of overt disease ^28^. Similarly, mice lacking JAM-A, a key TJ structural component, or the muscle myosin IIA heavy chain (NM IIA) showed increased intestinal permeability but only low-grade colonic inflammation and normal epithelial architecture ^29, 30^. Finally, zonulin transgenic mice constitutively producing high levels of zonulin showed increased gut permeability but not pathologic phenotype ^31^. Interestingly, zonulin transgenic, JAM-A ^−/−^, and NM IIA ^−/−^ mice all showed increased susceptibility to chemical-induced colitis ^29– 31^. Together, these data suggest that gut permeability may contribute to the development of several CIDs provided that additional genetic components regulating immune response and an imbalanced microbiome are coexistent. Indeed, there is growing evidence that the additional and mutually influenced elements of the triad of gut permeability, immune system, and gut microbiome—together with genetic predisposition and exposure to environmental triggers—make the “perfect storm” for CIDs development.

## Challenges in measuring zonulins with currently commercially available enzyme-linked immunosorbent assays

Several groups, including ours, have been questioning what exactly the commercially available zonulin enzyme-linked immunosorbent assay (ELISA) kits measure ^22, 32^. Using different approaches, both we ^22^ and others ^32^ identified complement C3 as the top match protein identified by the antibodies used for the ELISA. However, complement C3 is most likely an unspecific product overshadowing the real targets. Indeed, the respective ELISA kits did not detect any complement C proteins obtained from different suppliers when tested under native and denatured conditions, as well as when C3 was spiked in serum ^22^. Also, the same kit did not detect recombinant zonulin, mature HP1, or mature HP2 ^22^. Given the additional mass spectrometric hits reported ^22, 32^, a few proteins stand out, although, without further validation, these data need to be interpreted with caution, since only more abundant proteins may be identified by mass spectrometric analysis, while the protein(s) of interest belonging to the zonulin family may be in low abundance in serum samples and therefore not identifiable with this approach. Keeping this in mind and the fact that the protein(s) of interest should be in the roughly 50-kDa range, combined Western blot analysis and ELISA test confirmed that the polyclonal antibodies raised against the zonulin-derived synthetic peptide AT1001 and used in the Immundiagnostik kit (Immundiagnostik AG, Bensheim, Germany) detect properdin among other proteins ^22^. However, when purified proteins/peptides, including the AT1001 peptide used to raise the polyclonal antibodies (internal control), were tested by ELISA, they were highly underestimated by the test. One possible explanation for these results is that zonulin as both pre-HP2 and properdin is not the main target detected by the ELISA; however, the fact that even AT1001 was underestimated seems to suggest that this hypothesis cannot entirely explain these results. Alternatively, it is possible that tertiary and quaternary (multimers) structure arrangements present in sera samples but not in recombinant proteins are necessary in order to properly detect any zonulin member by this ELISA. Given these results, it is likely that the commercially available ELISAs detect one or more members of the zonulin family that have not been discovered yet.

## Alterative tests to measure gut permeability

With the appreciation that gut microbiome composition/function can affect intestinal permeability and vice versa and that loss of gut barrier function allows passage of endotoxin from gut lumen to systemic circulation, there are additional tools to be considered to monitor intestinal permeability. The presence of cytotoxic bacterial products in serum can be evaluated by using IgA/IgM responses to sonicated samples of common Gram-negative gut commensal bacteria, and assays of serum lipopolysaccharides (LPSs) and other bacterial toxins, including cytolethal distending toxin subunit B, provide good methods to screen for increased gut permeability in combination with IgM levels to zonulin and measuring gut dysbiosis ^33^.

## Role of zonulin pathway in specific chronic inflammatory disease

Zonulin has been implicated in many CIDs ( Table 1). Independent from the CIDs considered, the steps leading to break of tolerance and subsequent development of CID seem to be similar ( Figure 1). Below, we will review some of the CIDs that have been associated with dysregulation of the zonulin pathway, and a more complete list of diseases and related references is presented in Table 1.

**Table 1.** Chronic inflammatory diseases in which zonulin has been linked as a biomarker of gut permeability.

Disease	Model	References
Aging	Human	37, 38
Ankylosis spondylitis	Human	39
Attention deficit hyperactivity disorder	Human	40
Autism	Human	41, 42
Celiac disease	Human	15– 20, 23– 27, 43– 48
Chronic fatigue syndrome/myalgic encephalomyelitis	Human	49
Colitis – inflammatory bowel diseases	Human	50, 51
Colitis	Mouse	52
Environmental enteric dysfunction	Human	53
Gestational diabetes	Human	54, 55
Glioma	Human	56
Glioma	Cell	57
Insulin resistance	Human	58
Irritable bowel syndrome	Human	59, 60
Hyperlipidemia	Human	61
HIV	Human	62– 66
Major depressive disorders	Human	67, 68
Multiple sclerosis	Mouse	69
Multiple sclerosis	Human	70
Necrotizing enterocolitis	Rat	71
Necrotizing enterocolitis	Human	72
Non-alcoholic fatty liver disease	Human	73– 77
Non-celiac gluten sensitivity	Human	53, 78
Obesity	Human	79– 87
Schizophrenia	Human	41, 88, 89
Sepsis	Human	90
Type 1 diabetes	Rat	91
Type 1 diabetes	Human	92, 93
Type 2 diabetes	Human	94, 95

### Aging

Aging is the result of a constellation of cumulative changes that are deleterious, progressive, universal, and thus far irreversible. Aging damage can occur at the molecular (DNA, proteins, and lipids), cellular, or organ level (or a combination of these). Recent scientific successes in rejuvenation and extending a lifespan of model animals give hope to achieve negligible senescence, reverse aging or at least significantly delay it. Many non-mutually excluding theories have been formulated to model the senescence process, including the free radical theory ^34^, the cellular senescence and apoptotic theory ^35^, the immune system theory of aging ^36^, and (pertinent to this article) the intestinal permeability and aging theory. Indeed, several reports in both animal models and humans link gut permeability to non-infective chronic inflammation and metabolic changes typical of the senescence process. In fruit fly, the increase in intestinal permeability leads to systemic metabolic defects and immune changes previously linked to aging and is the best predictor of imminent death, even more than the actual age of the insect ^96^. In humans, it has been reported that zonulin serum concentration is higher in older adults, is positively associated with concentrations of the pro-inflammatory cytokines tumor necrosis factor alpha (TNF-α) and interleukin 6 (IL-6), and intriguingly is negatively correlated with skeletal muscle strength and habitual physical activity ^37^. These results suggest that zonulin-dependent gut permeability is associated with both systemic inflammation and two key indices of physical frailty associated with aging. These results have been corroborated by more recent data generated in disease-free ultra-centenarians showing lower levels of serum zonulin and endotoxemia as instigator of inflammation compared with young patients with acute myocardial infarction ^38^, supporting the notion that, as in the fruit fly, gut permeability may impact lifespan expectations.

### Autoimmune disorders

***Celiac disease.*** Celiac disease (CD) is an autoimmune enteropathy triggered by the ingestion of gluten-containing grains in genetically susceptible individuals and can be reversed when gluten is eliminated from the diet. As mentioned above, indigestible fragments of gluten are able to bind CXCR3 and release zonulin ^27^. CD has been used as a model disorder to study the effect of zonulin since its involvement in the development and pathogenesis of the disease has been well documented ^15– 20, 23– 27^. Even if gluten can trigger zonulin release in both healthy individuals and CD subjects, the amount and duration of zonulin produced are much higher in the latter group, leading to a significant increase in gut permeability, as shown by the capability of the zonulin inhibitor AT1001 (now named larazotide acetate) to prevent the zonulin permeating activity both in *ex vivo* models ^43, 44^ and in a transgenic animal model of CD in which it prevented gluten-dependent inflammation and intestinal damage ^38^. Larazotide acetate has been tested in patients with CD, showing good safety and efficacy in preventing gluten-dependent inflammation ^45– 48^, and is now in phase III clinical trial.

***Type 1 diabetes.*** Type 1 diabetes (T1D) is an autoimmune condition caused by the destruction of the insulin-producing β cells of the pancreas, and the pathogenesis of this disease is still not fully understood. Several studies, in both animal models and T1D patients, have shown increased intestinal permeability to precede the development of T1D ^97, 98^. In a recent elegant study, it was demonstrated that loss of gut barrier integrity was actually the causal factor for the microbiota-mediated T1D ^99^ in susceptible mice, further supporting the critical role of the gut barrier–microbiome–immune system triad in the pathogenesis of CID. BioBreeding diabetes-prone rats, which spontaneously develop T1D, have increased small intestinal permeability which precedes the loss of tolerance to glucose by at least one month ^100^. Oral administration of the zonulin blocker AT1001 in these rats corrected the gut barrier defect and reduced the incidence of diabetes, suggesting a mechanistic role of the zonulin-dependent gut barrier modulation in the pathogenesis of T1D ^91^. The involvement of zonulin in T1D was confirmed in human studies showing that about 50% of patients with T1D have increased serum zonulin levels, some of them showing these changes in the pre-diabetic phase of the disease ^92^. Interestingly, a subset (about 25%) of healthy first-degree relatives of patients with T1D also showed increased serum zonulin ^92^. Similar data were generated in children at risk of T1D in which zonulin correlated with Glo-3A antibodies (a potential biomarker of the disease) in cases (at-risk children in the pre-clinical phase [positive auto-antibodies] or overt T1D) but not in controls (at-risk children negative for auto-antibodies) ^93^. Combined, these data suggest that zonulin may play a role in the pathogenesis of T1D in a subset of patients.

***Inflammatory bowel disease.*** Increased intestinal permeability has been shown to play a crucial role in the pathogenesis of inflammatory bowel diseases (IBDs) ^101– 105^. Arrieta *et al.*, using the IL-10 knockout colitis mouse model, showed increased small intestinal permeability that preceded the onset of the overt colitis that can be ameliorated by oral treatment with zonulin inhibitor AT-1001 ^52^. In humans, serum and fecal zonulin were found to be elevated in patients with active Crohn’s disease but not with ulcerative colitis ^50^. In a more recent study, serum zonulin concentration was found to be higher in both diseases, and an inverse correlation was observed between serum zonulin concentration and disease duration ^51^.

***Multiple sclerosis.*** In the experimental autoimmune encephalomyelitis mouse model of multiple sclerosis (MS), zonulin-dependent increased intestinal permeability was shown during the pre-clinical phase of neurological symptoms, suggesting a role for zonulin in disease development ^69^.

It has been reported that patients affected by MS show increased permeability of both the blood–brain barrier (BBB) and the intestine. A recent report showed that zonulin concentrations were significantly higher in MS patients showing a compromised BBB as shown by magnetic resonance imaging ^70^. Interestingly, baseline zonulin concentrations were associated with 1-year disease progression in progressive MS and closely mirror BBB breakdown in relapsing remitting MS. Considering these results, the authors concluded that zonulin may be responsible for the breakdown of both the intestinal barrier and the BBB in gut dysbiosis, thereby explaining how the gut–brain axis modulates neuroinflammation in MS ^70^.

***Ankylosing spondylitis.*** Ankylosing spondylitis (AS) is an inflammatory, autoimmune condition that typically begins in young adulthood but often gets overlooked or is incorrectly diagnosed as pain from a previous injury or aging. It is an underdiagnosed form of arthritis that creates inflammation in the spinal joints and causes chronic back pain and stiffness. Dysbiosis has recently been demonstrated in patients with AS but its implications in the modulation of intestinal immune responses have never been studied. By analyzing ileal biopsies from patients with AS, Ciccia *et al*. showed that the presence of adherent and invasive bacteria in the gut of patients with AS with the bacterial scores significantly correlated with gut inflammation ^39^. Impairment of the gut vascular barrier was also present in AS, accompanied by significant upregulation of zonulin, and was associated with high serum levels of LPS, LPS-binding protein, intestinal fatty acid–binding protein (iFABP), and zonulin ^39^. In *in vitro* studies, zonulin altered endothelial TJs while its epithelial release was modulated by isolated AS ileal bacteria. Furthermore, they provided evidence that bacterial products and zonulin influence monocyte behavior. Considering these results, the authors concluded that bacterial ileitis, increased zonulin expression, and damaged intestinal mucosal epithelial and endothelial barriers characterize the gut of patients with AS and are associated with increased blood levels of zonulin and bacterial products.

### Metabolic disorders

***Obesity.*** Obesity and its complications, including high cholesterol, type 2 diabetes (T2D), coronary heart disease, high blood pressure, and stroke, have been shown to be associated with chronic inflammation ^106– 108^ and frequently linked to alteration of the zonulin pathway, and more than 30 articles have been published on this topic. Several of these studies have shown elevated serum zonulin levels increased in obese versus non-obese subjects ^79– 86^, and there is evidence of a correlation between total bacteria and serum zonulin levels, suggesting that the gut microbiota may cause increased zonulin levels, and subsequent abnormal gut permeability to endotoxin and ultimately micro-inflammation has been reported in obesity ^106^. A recent report also showed that zonulin serum correlates with total calorie, protein, carbohydrate, sodium, and vitamin B _12_ intake in obese women, and *Ruminococcaceae* and *Faecalibacterium* were more abundant in the low-zonulin group, suggesting that butyrate-producing gut bacteria such as *Faecalibacteria* could decrease gut permeability by decreasing zonulin levels and lower inflammation ^87^.

***Other metabolic disorders.*** Additional evidence suggests that zonulin is associated not only with obesity but also with its metabolic complications, including insulin resistance ^58^, non-alcoholic fatty liver disease ^73– 77^, gestational diabetes ^54, 55^, hyperlipidemia ^61^, and T2D ^94, 95^.

### Intestinal diseases

***Irritable bowel syndrome.*** Increased gut permeability has also been linked to the pathogenesis of irritable bowel syndrome (IBS) ^59^. Specifically, patients with diarrhea-associated IBS showed increased serum zonulin levels ^60^ and involvement of the protease-activated receptor 2 (PAR2) ^109, 110^, the zonulin target receptor ^23^.

***Non-celiac gluten sensitivity.*** Non-celiac gluten sensitivity (NCGS) is a clinical entity triggered by gluten as in CD but without autoimmune enteropathy ^111^. It has been shown that patients with NCGS may have increased serum zonulin levels and increased intestinal permeability following gluten exposure ^60, 78^.

***Environmental enteric dysfunction.*** Environmental enteric dysfunction (EED) is a chronic disease affecting mainly the proximal intestine. It is characterized by loss of barrier function, bacterial overgrowth in the small intestine, and low-grade intestinal inflammation leading to small intestinal villous atrophy that, in some aspects, resembles CD enteropathy. The potential developmental consequences of EE/EED can be devastating to the full physical and neurocognitive development in one third of the world’s children growing up in impoverished areas. It was recently reported that serum zonulin levels and other markers of barrier dysfunction were correlated with stunted growth in patients with EED ^53^.

### Cancer

There is growing evidence in the literature that Ag trafficking can also be involved in the immune component leading to the onset of a variety of cancers. Here are reported those conditions in which zonulin as a biomarker of epithelial and endothelial permeability has been associated with cancer.

***Glioma.*** Zonulin has also been shown to be involved brain tumors, mainly gliomas ^56, 57^. Increased zonulin expression of zonulin in gliomas correlated with the degree of malignancy and degradation of the BBB ^56^. *In vitro* studies on a glioma cell line showed that zonulin was expressed in high amounts compared with non-glioma control cells ^57^. Additionally, zonulin has been shown to induce transmigration of neuronal progenitor cells across the BBB ^57^.

***Hepatocellular carcinoma.*** Hepatocellular carcinoma (HCC) globally ranks fifth for incidence and third for mortality among all malignant tumors. Although some progress has been made in exploring the pathological mechanisms and interventions of chronic liver diseases, there are still no effective biomarkers for the prediction and prevention of the progression of chronic liver diseases. Wang *et al*. recently reported that serum zonulin levels were significantly higher in patients with HCC compared with patients with liver cirrhosis or chronic hepatitis B or healthy subjects ^112^. Moreover, the zonulin levels were increased in the advanced stage of liver cirrhosis and HCC.

### Neuroinflammatory diseases

Loss of gut barrier function (evaluated by serum IgG/IgA/IgM responses to occludin and zonulin and IgA responses to actomyosin) with subsequent increased serum levels of microbiota-derived molecules (assayed by testing serum lipopolysaccharides and bacterial toxins, including cytolethal distending toxin) and activation of the immune system (increased cytokines production) leading to neuroinflammation has been described in many neuroimmune disorders, including chronic fatigue syndrome, autism spectrum disorder (ASD), major depressive disorders (MDDs), and schizophrenia ^32^.

***Autism spectrum disorder.*** Increased serum zonulin that positively correlated with the Childhood Autism Rating Scale score has been reported in children with ASD ^41^. In autism, as in other neuroinflammatory disorders, changes in zonulin-mediated gut permeability lead to pro-inflammatory status characterized by increased levels of mucosal pro-inflammatory cytokines (IL-5, IL-15, and IL-17) and decreased anti-inflammatory cytokines (transforming growth factor beta 1, or TGFβ1) detected mainly in ASD children experiencing gastrointestinal (GI) symptoms ^42^.

***Schizophrenia.*** Increased plasma IgA/IgM responses to Gram-negative bacteria have been reported in deficit schizophrenia ^112^ indicating leaky gut and gut dysbiosis. These results were confirmed more recently by Maes *et al*., who reported that the ratio of IgM to zonulin + occludin/talin + actin + viculin was significantly greater in patients with deficit schizophrenia than in those with non-deficit schizophrenia and higher in patients with schizophrenia than in controls and was significantly associated with increased IgA responses to Gram-negative bacteria ^88^. IgM responses to zonulin were positively associated with schizophrenia (versus controls), whereas IgM to occludin was significantly associated with deficit schizophrenia (versus non-deficit schizophrenia and controls) ^88^. The results show an upregulated paracellular pathway with breakdown of the tight and adherens junctions and increased bacterial translocation in deficit schizophrenia, suggesting their mechanistic role in causing neuroinflammation typical of the disease. These data were recently confirmed by our group ^89^.

***Major depressive disorders.*** Gut dysbiosis consistent with pathophysiological gut metagenomic signatures (upregulation LPS biosynthesis genes and deleterious metabolism of mood neurotransmitter pathways and host intestinal protective glycosaminoglycan mucins) different when compared with normal controls has been described in MDD ^67^. Parallel to these changes, subjects affected by MDD showed increased plasma levels of LPS, zonulin, and FABP2 ^67^. Additionally, it has been demonstrated that, in patients with MDD, zonulin-mediated increased gut permeability causing increased bacterial translocation leads to marked alteration in circulating monocytes, with an expansion of the intermediate subset with increased frequency of IL-1β– and IL-6–producing cells. These changes are associated with a systemic pro-inflammatory state characterized by the enhanced serum TNF-α and IL-1β levels compared with those in the healthy controls ^68^.

***Chronic fatigue syndrome or myalgic encephalomyelitis.*** Chronic fatigue syndrome/myalgic encephalomyelitis (CFS/ME) is an illness characterized by profound and pervasive fatigue in addition to a heterogeneous constellation of symptoms. The etiology of this condition remains unknown; however, it has been suggested that enteric dysbiosis is implicated in the pathogenesis of CFS/ME ^49^. In a systemic review, the microbiome composition of patients with CFS/ME was compared with that of healthy controls, showing statistically differences in some studies but with inconsistent findings in the studies considered ^49^. Nevertheless, evidence of loss of barrier function in CFS/ME ^32^ is consistent with the overall theme of mutual influence between gut dysbiosis and intestinal barrier function.

## Conclusions

Besides genetic predisposition and exposure to environmental triggers, the pathogenesis of a variety of CIDs seems to involve mutually influenced changes in gut permeability/Ag trafficking, immune activation, and changes in composition/function of the gut microbiome. Zonulin is a modulator of both epithelial and endothelial barrier functions and its role in health and disease remains an object of active research. Gut dysbiosis may cause the release of zonulin leading to the passage of luminal contents across the epithelial barrier causing the release of pro-inflammatory cytokines that themselves cause increased permeability establishing a vicious loop leading to massive influx of dietary and microbial Ags triggering the activation of T cells. Depending on the host genetic makeup, activated T cells may remain within the GI tract, causing CID of the gut (IBD, IBS, CD, and EED), or migrate to several different organs to cause systemic CID. The effect of the zonulin inhibitor larazotide acetate in mitigating inflammation both in animal models and in human clinical trials not only confirms the pathogenic role of zonulin in many CIDs but also opens the possibility of targeting gut permeability in a variety of CIDs in which a pathogenic role for zonulin has been hypothesized or proven.

## Abbreviations

Ag, antigen; AS, ankylosing spondylitis; ASD, autism spectrum disorder; BBB, blood–brain barrier; CD, celiac disease; CFS/ME, chronic fatigue syndrome/myalgic encephalomyelitis; CID, chronic inflammatory disease; EED, environmental enteric dysfunction; ELISA, enzyme-linked immunosorbent assay; GI, gastrointestinal; HCC, hepatocellular carcinoma; HP, haptoglobin; IBD, inflammatory bowel disease; IBS, irritable bowel syndrome; IL, interleukin; JAM, junctional adhesion molecule; LPS, lipopolysaccharide; MASP, mannose-binding lectin-associated serine protease; MDD, major depressive disorder; MS, multiple sclerosis; NCGS, non-celiac gluten sensitivity; T1D, type 1 diabetes; T2D, type 2 diabetes; TJ, tight junction; TNF-α, tumor necrosis factor alpha; ZO-1, zonula occludens 1
